# A Narrative of the Treatment Experienced by a Gentleman during a State of Mental Derangement; Designed to Explain the Causes and the Nature of Insanity, and to Expose the Injudicious Conduct Pursued Towards Many Unfortunate Sufferers under That Calamity

**Published:** 1841-01

**Authors:** 


### ART. V.

1. *A Narrative of the Treatment experienced by a Gentleman, during a state of Mental Derangement; designed to explain the Causes and the Nature of Insanity, and to expose the injudicious conduct pursued towards many unfortunate sufferers under that calamity.—London,* 1838. 8vo, pp. 278.

2. *A Narrative of the Treatment experienced by a Gentleman, during a state of Mental Derangement; designed to explain the Causes and the Nature of Insanity, and to expose the injudicious conduct pursued towards many unfortunate sufferers under that calamity.* By JOHN PERCEVAL, Esq.—*London,* 1840. 8vo, pp. 430.

3. *Aphorisms on the Treatment and Management of the Insane; with Considerations on Public and Private Lunatic Asylums, pointing out the errors in the present system.* By J. G. MILLINGEN, M.D., Surgeon to the Forces; late Medical Superintendent of the County of Middlesex Pauper Lunatic Asylum at Hanwell, &c. &c.—*London,* 1840. 8vo, pp. 202.

4. *Bemerkungen über den Umgang mit Geistkrankheiten, für diejenigen die mit ihnen in Berührung kommen.* Von J. F. LEHMANN, Inhaber einer Privat-Heil-Irrenanstalt auf dem Wedding bei Berlin.—*Berlin,* 1837. 8vo, pp. 51.

*Remarks on the conduct to be observed towards Lunatics by those who are in charge of them.* By J. F. LEHMANN, Proprietor of a Private Lunatic Asylum at Wedding, near Berlin.—*Berlin,* 1837.

5. *Du Danger des Rigueurs Corporelles dans le Traitement de la Folie.* Par le Docteur BLANCHE, de Montmartre, Médecin des Hôpitaux de Paris (Section des Aliénés).—*Paris,* 1839. 8vo, pp. 63.

*Of the Danger of Corporal Severities in the Treatment of Insanity.* By Doctor BLANCHE, of Montmartre, Physician to the Hospitals of Paris (Section of Lunatics).—*Paris,* 1839.

6. *The Fifty-fifth Report of the Visiting Justices of the County Lunatic Asylum, at Hanwell; and Second Report of the Resident Physician* (Dr. CONOLLY).—*London,* 1840. 8vo, pp. 128.

IT is not often, we imagine, that opportunities present themselves of obtaining such detail of the thoughts, feelings, and propensities of a lunatic, or of an individual who has laboured under insanity as can be relied upon. Disinclination to look back upon so unhappy a period often prevents all allusion to it. Some remaining feebleness or irritability of mind also often renders it impossible to receive such statements as are made, without much abatement. The impressions of madness are sometimes fleeting, like those of dreams. The instances, above all, are few in which individuals of known vigour of mind have sustained an attack of insanity, and recovered so far as to refer to it with composure, as they would to any other kind of calamity; or to review the visitation philosophically, as a psychological phenomenon at once curious and terrible. No greatness of intellect enables its possessor to contemplate its diminution with resignation. Other evils may be borne, but this is the compendium of all possible evils; and to have suffered it once, if only for a time, produces too painful a conviction of the insecurity of mental power for any mind to reflect upon without dread.

The celebrated Robert Hall is described by one of his biographers, after his first attack of insanity, which was but of six weeks' duration, as pale and emaciated, and his spirits broken by "severity and harshness," and as discovering to his friend, "with a heart full of injuries, all the secrets of his prison-house, with his feelings and usage at the time." We may learn something of these feelings, when we find such a man complaining that they "took away his watch" in the asylum to which he had been sent; but Robert Hall's own words have a claim to quotation:

"'Sir, they took my watch, and confined me in a place which overlooked the ward in which were a number of pauper lunatics practising all manner of ludicrous antics. Sir, this sight was enough to make me ten times worse; they were as mad as March hares; I was at times quite insensible. I don't believe Dr. ——— was aware how I was treated by a lazy keeper. Do you know, sir, to save himself a little time and trouble (being winter), the fellow came at five o'clock and fastened me down upon my bed, where I could not stir either hand or foot, till about eight o'clock the next morning. During this time I had many lucid intervals; he had no business to leave me, sir, so long, but it was to enable him to go away sooner. You cannot conceive the horror of my situation, when I found myself perfectly sensible.......Now, sir, I hope, if ever I am taken ill again, you will use all your influence to prevent my being sent there a second time. It is a very mistaken notion that severity is requisite. *Mild treatment, with proper restraint and kindness,* is all that is necessary in such cases. There is nothing so beneficial as private confinement, with proper medical attendance, to prevent abuses by unfeeling keepers.' Then, with great energy, he repeated the request, 'I must beg, sir, you will do all in your power to prevent such treatment in case of a relapse.'" (Green's Reminiscences of the Rev. Robert Hall.)

From such a man, these are affecting words; and those we are about to quote are even more so. Being complimented on a sermon he preached soon after his recovery, his observation was:

"'O do not say anything about it, sir; I shall never be the preacher I was. I find I have lost the principal faculty that distinguished my preaching, which was imagination; you know that was my forte, sir; all my imagination has been overstretched. You, with the rest of my friends, tell me that I was only seven weeks in confinement, and the date of the year corresponds, so that I am bound to believe you, but they have appeared to me like seven years. My mind was so excited, and my imagination so lively and active, that more ideas passed through my mind during those seven weeks than in any seven years of my life. Whatever I had obtained from reading or reflection was present to me; I had all my ideas at my fingers' ends, and could bring them to bear upon any subject.'" (Ibid.)

That Robert Hall's painful representation of his treatment in his first attack was correct is rendered probable by the very different account he gave of the mode in which he was treated in a second attack of his lamentable malady. His slightest observations derive importance from his character, from the known powers of his understanding, and from the circumstance that so accomplished a friend as Sir James Mackintosh ventured not only to correspond with him on the subject of his insanity, but addressed to him, in reflections drawn from the greatness of the sufferer's intellectual character, the noblest topics of consolation ever applicable in such circumstances, and which were never before so eloquently applied.

The volumes placed first in the list at the head of this article, although the production of an individual of powers very inferior to Hall, are still not only very singular compositions, but far from being uninstructive. We must, however, chiefly restrict our observations to the first volume: the second, which followed it after a considerable interval, has more of repetition and useless detail, unmingled with the consistent and touching reflections which occur in the former. The chief value of the narrative, and that which strongly recommends it to all who undertake to manage lunatics, is that it explains the imaginary reasons for many of the actions of the insane, otherwise not to be divined; and that it records the impression made on the mind, even in its disturbed state, by the treatment administered. The last we consider to be a point of the extremest value, and one which has never yet received adequate attention.

We shall not dwell on the innumerable details of which this melancholy story is composed; yet it is impossible to pass over the author's preliminary history, and not to lament that a young gentleman of good connexions, of cultivated mind, of tender conscience, and of an excitable imagination, should have been left to wander unguarded among the most ignorant and besotted male and female bigots that the kingdom was ever infested with. To mingle with these people, he left his college (Oxford); and his mind, already a little off its balance, was soon overthrown by the wildness, ignorance, and superstition to which it was recklessly exposed. At Port Glasgow he was privileged to hear a "manifestation of tongues," by which his unsettled brain was so agitated that he discoursed on religious matters at night in the traveller's room at the inn, over whiskey-toddy, with a "kind of frolicsome squire or laird," who felt or pretended to feel a sudden contrition; upon which the author of the book *sung* words of comfort to him, in an inspired way, and the squire, "awed into compunction," rose up and kissed him.

After this, his progress was rapid. When a clergyman was preparing to preach, and "the rest of the congregation were at peace," he was led, he says, to open his mouth, "and sing a part of a psalm." The clergyman descended from the pulpit, and tranquillized him; but his chief adviser touching this essay appears to have been a lady, who caused him to see his error by the clear method of showing that "we are not to speak altogether, but to command the spirits." Removal from such society to a lunatic house would have been a change very much for the better. But it was yet long before he was placed under proper protection or salutary guidance. Going to Dublin in this zealous and heated frame of mind, on the principle, one would think on which Hamlet was to be sent to England, his worst delusions were strengthened by the religious excesses of those with whom he associated. In a state of such excitement that he was continually attempting "utterances and singing;" conceived a red handkerchief given to him was blood, and a token of ill; and even wished to put his hand into the fire, persuaded that he might draw it out unhurt; he was still, apparently, left to his own management; and thus, by too common an error, the precious period of incipient or of active disease was neglected. In such cases, the first neglect ever ushers in a long train of hurtful modes of subsequent treatment. The period of prevention being past, the poor lunatic is next pretty sure to be exposed to a prolonged punishment, for such it is, for evils which vigilant care might probably have prevented.

All the horrors of insanity soon came upon the unfortunate subject of the book. He awoke in the night with dreadful impressions; he heard a voice charging him with "disobedience to the faith, in taking the medicine overnight," by which he had rendered his salvation extremely difficult, by its effect upon his "spirits and humours." He was told that he could only be saved by being changed into a spiritual body, by a great fight, to take place in his mortal body between satan and Jesus. A spirit came upon him and guided his actions; he was placed in a fatiguing attitude, resting on his feet, his knees "drawn up and on his head," and made to swing his body from side to side without ceasing. Voices, and sounds as of clanking iron, and the breathings of great forge bellows, and the force of flames, were heard within and without him. After a long struggle, he considered his salvation to be completed; and that he was to proceed through the world "as an angel," and "in an extraordinary way, and by singing." At this time, however, a military friend, who appears to have seen that all this was less reasonable than it might appear to the zealots by whom the poor patient had been surrounded, interfered, and placed him under medical care.

The lamentable workings of a perverted piety were now most painfully endured. When he found his going out prevented by a stout man-servant on the landing, placed there by a physician, he declared himself a prophet; seized one of the man's arms, and desired it to wither; and, the effect not following, did not still mistrust "the doctrines," but thought himself mocked by the Almighty for his disobedience of them; and for not having waited for the spirit to guide his action when the word was spoken; and because he had seized the man's arm with the wrong hand. At one time he was desired by "voices" to sing, at another to pray, and by some maladroitness he misplaced every injunction, and as surely received, from "the voices," the most cutting and insulting reproaches. At last, in one of his mental conflicts, "hunted in every direction," his patience, he relates, gave way, and he "mentally cursed the Holy Trinity." He adds, "a cutting sense of my ingratitude, and deep grief, followed with mute despair." These passages are calculated to excite the sincerest pity. So much conscientious feeling, mingled with such wild disorder of the reason, called for the most judicious interposition; for the most delicate and temperate interference; for all that kindness could dictate, regulated by all that calm good sense could approve. It does not appear to us that it was the author's good fortune to meet with this kind of treatment. We know with what reserve the statement of an unhappy man whose reason has once been fully dethroned must be received concerning the circumstances which took place in the dark period of his intellect; but there are circumstances related in this work which bear the stamp of truth upon them, so as not to be mistaken, and which not only show to what degrading discipline he was subjected, (for that, being common, is of no account,) but how keenly, even in his abject mental state, he suffered from useless or, as we deem, from ignorant and hurtful inflictions, from coarse and improper hands.

Little do those know who look with contempt or ridicule on the antics of the lunatic, what deep and serious meanings are sometimes attached to them, in his disordered mind. He is seen to limit his walk with undeviating scrupulosity to two or three flag-stones, or he takes up his position day by day in one corner, or he raps his forehead smartly with his palm some scores of times, and with unheard-of grimaces. For all this he may be scolded, or jostled about, or beaten; but this sagacious treatment has no influence on his prepossessions : it does but add to distraction a sense of burning insult. The lunatic becomes ferocious, and he is put into restraint; his voice becomes elevated, he raves, he despairs. All this seems in the ordinary course of things; and he dies as much a riddle as he lived.

Listen to our unfortunate author. The *voices* informed him that his conduct was owing to a spirit of mockery and blasphemy having possession of him, and that he must *redeem himself* and rid himself of these foul spirits. There was certainly something inconvenient in the mode in which he expected to effect this exorcism.

"The way in which I was tempted to do this was by throwing myself on the top of my head backwards, and, so resting on the top of my head and on my feet alone, to turn from one side to the other until I had broken my neck. I suppose by this time I was already in a state of feverish delirium, but my good sense and prudence still refused to undertake this strange action."

Who can fail to see that there was still an opportunity of corroborating that sense and prudence, and of moderating that feverish delirium? But by the side of this accomplished afflicted man there was but a stout man-servant, whose weapons were those usually brought to bear on the insane, mere brute force and bodily restraint. "Had he not been there," says the patient, "I might by that time have been sound asleep." Yes, poor sufferer! or if, instead of the stout servant, there had been some kind physician or some Sister of Charity by thy side, or even some one of thine own rich and well-sheltered, and not unkind family, thy feverish brow might have been cooled, thy faltering reason steadied, and thy neck-breaking summersets driven out of thy perturbed head. The man-servant adopted the only alternative in his power: he procured another stout man, and, together, they forced the poor neck-breaker into a strait-waistcoat, and tied his legs to the bed-posts, and then all was quite comfortable. "I had no design," says the patient, looking back on these conflicts," to destroy myself; but I have often conjectured since, that God in his mercy may have meditated my self-destruction to save me from the horrors he foresaw preparing for me: they were great and intolerable, shocking in themselves, more shocking in my abandonment. I awoke from them as from the grave, to be cut off from all my tenderest ties." Whatever may be thought of these sentiments, there can be but one opinion of the treatment, of which the bare recollection suggests them.

Bound and helpless in his bed, the patient lay, as usual, much disinclined to speak; those about him little dreaming wherefore to them he uttered not a word. But the patient knew wherefore; and those who look and talk heedlessly at the bedsides of lunatics, disabled from expressive attitudes by strait-waistcoats, may not uselessly remember his reasons. "I was unable to explain myself," he says," "because Dr. —— was reputed to be an Unitarian, and therefore I conceived it impossible to make him credit the supernatural voices and agency under which I acted. His companion seemed so stupid, and so like a man of the world of a common and vague stamp of mind, that I thought it perhaps still more hopeless to address him." Two things may be gathered from these hasty remarks of the poor man in bonds; first, that the insane may yet require and be consoled by a manifestation of reasonable sympathy with their sorrows; and secondly, that men of the world, of a common and vague stamp of mind, are by no means qualified for this line of practice. "They remained," he adds, "about five or ten minutes on the left hand side of my bed, and then went away." He says he afterwards learned that the *second* doctor (he of the vague aspect) was a celebrated lunatic doctor, to whom alone he ascribes the being confined to his bed "in nearly one position for several days together, tied hand and foot, in a strait-waistcoat, and in a small and close room." His first physician, he thinks, would have reasoned and acted differently. "He would have said, 'whatever harm may be in him, or may arrive to him from his complaint, it cannot be greater than what will certainly happen if he be confined.'" We entirely agree with the lunatic; and we read with shuddering the natural detail of the results of a neglect of such reasonable considerations.

"My need of wholesome exercise and occupation was denied. My idleness of body and mind left me at the mercy of my delusions; my confined position increased or caused a state of fever, which brought on delirium, and they kept drenching my body to take away the evil which their system was continually exciting, and which ultimately triumphed completely over me. My want of exercise produced a deadly torpor in the moral functions of my mind, combined with the ruin of my spirits by their diet and medicines."

Now, although the diet and the medicines might be innocent of ruining the spirits of the patient, what can be said in defence of that denial of exercise of body and mind, that confined position, and that unnatural drenching thus made necessary? Where is the man who could lie thus constrained, heated, disregarded, and drenched for days and nights, without "torpor in the moral functions of the mind, or without absolute frenzy?" Physicians think too little of these things; but out of the mouths of the insane wisdom cries to them, and they would do well to hear it and profit by it. Poor fellow! his simple language has an irresistible force in it:

"Since boyhood I had never been confined to my bed for more than two or three days, nor to my room for so much as a week together, and, on an average, had never had less daily than three hours active exercise. Now, after a fortnight's confinement to my room, I was fastened on my bed, with the liberty of my arms and legs denied to me."

But perhaps there were reasons for this. By the patient's account there were two: One was, he confesses, that when set at liberty he strove to renew his summersets; a triumphant reason in the eyes of many lunatic doctors, perhaps, but not in ours. The other reason was, *to get water, for which he longed;* and in which he says, "I think I succeeded once." He had another reason, also; namely, that he wished to look out of the window, thinking all his family were there to receive him. Under this enlightened treatment he came, not unnaturally, to think himself "the one only being to be eternally damned, alone, in multiplied bodies, and in infinite solitude and darkness, and torments." We can scarcely wonder at this; doomed to be fastened down, kept hot, and debarred from cooling drinks, in fever and in madness, night and day.

Yet all this time he was not fortunate enough to be insensible. The mind was working, but unaided; struggling, but without a friend. Let any physician in his senses read the following passage, and ask himself whether the patient's delusions might not have been in some degree relieved by allowing him to look out of the window, as he so much desired, and by calmly and soothingly taking the opportunity, which all who know much of lunatics know is of occasional but of most precious occurrence, of sapping his insane belief, and confirming his sane but wavering doubts. By this method, also, all the exhausting and irritating struggle, and perhaps all its consequences, might have been prevented.

"For I had a species of doubts; but no one who has not been deranged can understand how dreadfully true a lunatic's insane imagination appears to him, how slight his sane doubts. But I was not permitted to reach the window, and I was tied down again in bed; then my usual delusion came upon me, that I was gifted with the power of an elephant to break my bands; and when I tried and found how futile were my efforts, I was told I did not choose to use the strength I had, from cowardice, or ingratitude, or laziness. On one occasion, I remember, after my brother had come to attend me, a spirit came to me whilst I was lying on my back, fatigued with my efforts to break the strait-waistcoat by forcing out my arms and elbows laterally, and said, '*Use thy strength, I will show you how to do it.*' The spirit then guided my arms and my hands, and with my fingers *sought and scratched the seams of the waistcoat sleeves*, soon loosened them, and I began tearing the seams asunder. The noise of the rending asunder, however, soon aroused my attendant; my strait-waistcoat was taken off, and my arms were crossed over my stomach in two heavy, hot, leather arm-pieces, which were not taken off from me for good until I reached England. I feel thankful now for their removal."

Think of these things, advocates of restraint! Ask yourselves whether the evidence of those who have suffered these inflictions is not more to be depended upon, as to the effect of them, than the flippant sayings of men of the world, vague and common; and who, never missing their daily comforts, their dinner, their club, or their seat in the theatre, tell you, as Dr. Millingen tells us in his Aphorisms, that the sovereignest thing on earth is a strait-waistcoat for an inward bruise; or, at least, that attempts to do without restraint are "speculative and peculative," and lead to a "useless waste and dilapidation of property." They forget, good easy men, that this destruction might be prevented by efficient keepers in sufficient numbers. But *this*, also, is expensive; ruinous in private houses, and not to be thought of for paupers. It is very true, but it is also expensive to keep lunatics alive at all; and if cheapness is to be the criterion of excellence then let them all die, relieve the rich families and relieve the much oppressed parishes, and put an end to the fond speculation and peculation of treating them humanely.

There is something amusing, but not uncommon, in the history of recent attempts to mitigate the treatment of lunatics by abolishing these personal restraints. First, it is sneered at as a thing impossible; then it is reflected upon as not only visionary but dishonest—not merely speculative, but, by ingenious alliteration, peculative too; then its perfect practicability is granted, but its humanity gravely denied. At length it is claimed as a virtue long practised, and in each asylum the strait-waistcoat is disavowed, and the muff sacrificed to the public opinion; these disappear, dive to the basement-story, not without inconsistent aspersions cast on those who have forced them to this exercise of virtue. In the mean time we trust, not too confidently, but still trust, that the abolition of cruel and useless methods of personal coercion makes a steady and quiet progress; reflecting on none save by contrast, and challenging none save by example.

Those who have long floated, by the help of day and night restraints, in a sea of madhouse troubles, are likely to sink when such bladders are withdrawn. A troublesome patient brings them to their wit's end! To discontinue common bonds and manacles calls for vigilance, for forethought, and for all the ingenuity of preventive treatment, assisted by every practical means of diverting the mind from its haunting delusions. These things require reflection; and, as reflection is more troublesome than the imposition of restraint, those unused to reflection will accord no smile of favour to them; yet the very victims of their indolence see what might be done. Even the author of the work before us, on his restless delusive pillow, manacled hand and foot, and in a whole fortnight only once, and that by a rare accident and happy sally, obtaining water to drink; even he, in a situation not favorable to tranquil meditation, could imagine, and, being recovered, can recollect what might have been better than all this. Again we quote his own simple words:

"It may be asked, what course I would have had pursued towards me, seeing there was such evident danger in leaving me at liberty? I answer, that my conduct ought to have been tried in every situation compatible with my state; that I ought to have been dressed, if I would not dress myself; that I should have been invited to walk up and down my room, if not quietly, in the same confinement as in bed; that, whilst implements that might do me hurt were removed, pens, pencils, books, &c. should have been supplied to me; that I should have been placed in a hackney-coach, and driven for air and exercise towards the sea-shore, and round the outskirts of Dublin. Few can imagine the sense of thirst and eager desire for freshness of air, which the recollection of that time still excites in me. I do not recollect water having been presented to me; if it was I systematically refused it, like everything else, and it was not forced on me like the medicine and broth. If I recollect correctly I got some water after my brother's arrival, and he also brought me once some grapes, a few of which I ate in spite of my false conscience, and God knows how refreshing they were."

The injudicious language addressed to lunatics, even by those who have the care of them, has often struck us with surprise. It shocks us to see a poor demented biped standing before his doctor, whilst the said doctor enumerates the said biped's faults, errors, weaknesses, and peccadilloes, with marginal remarks on the faults of his father, the errors of his mother, the weaknesses of his grandmother, and the peccadilloes of all his other relatives. We have repeatedly seen those who have once attempted suicide thus reminded of the attempt, and their memories refreshed as to all the particulars, and even shameful crimes spoken of as having been laid to the lunatic's charge, until we have observed all the blood in the supposed insensible biped mounting up into his conscious face, and have expected, and almost hoped, that he would cut his reviler's story short by knocking him down. Smaller offences against the lunatic are perpetually committed; such as stirring him up on his mad subject; asking him questions, the replies to which are likely to manifest his delusions; staring at him as at a wild animal, or laughing in his face. The folly of all this is very great, but it renders a visit to a lunatic asylum amusing to people of small benevolence. Such a visit, by exhibiting the composure of weak minds under mild parental care, excluding all hurtful excitement, ought to gratify the noblest feelings of humanity, rather than to divert idle visitors, who come to see the asylum to-day because they visited the wild Indians yesterday. The subject of the narrative which now occupies us remarks of a relative, that he replied to him, when he spake according to his delusions, in an "ill-judged tone of levity, and as if speaking to a child, ridiculing the idea;" by which, he says, "his hopes of being comprehended were blighted." He reflected, he says, that his relatives should have remembered that it could be no light matter which had so changed him; and he is of opinion that if he had been reasoned with, the possibility of his inspirations admitted, but their probability questioned, he should have been rescued from his "dreadful situation and saved from ruin."

After a time, however, he found himself at liberty to get up and dress, and to help himself to breakfast. He was taken down stairs, put into a hackney-coach, driven to the quay, and put on board a Bristol packet, with his relative and the stout man-servant. Having a fancy for walking about in the packet (not very unnatural after a fortnight's attachment to the bedposts), he was made to go to bed again; "which, God knows," he says, "I had had enough of." He soon became again the sport of the wildest delusions; many of which are detailed, and some of which are exactly like long and vivid dreams. One of his unfortunate fancies was, that it was necessary for him to devote himself to death to save the ship; and, in such difficult circumstances, this notion was evidently incompatible with his being at liberty. On arriving at Bristol another doctor was introduced to him, who, as usual, ordered him *to bed.* "I would have given my hand to remain up; my bed was a scene of horrors to me. However, I made no reply, and to bed I went." Now, this is just the case to support the views of those who advocate the comfort of being strapped down in bed. What would you do? say they. Would you, instead of confining the patient by a gentle strap, have two strong men to sit by him and frighten him with their looks? We would do neither; neither have two stout servants instead of one, nor yet adopt the asserted milder scheme of strapping down the patient to his bed. What, then, *would* you do? We should *let him sit up.* Upon what possible ground of reason would you, *we* would ask, force a poor creature into his bed who would "give his hand" to remain up? Where is the humanity you boast of in thus confining him, by strap and chain, to a "scene of horrors." Such treatment is opposed to the first principles of the curative art in relation to irritated minds. It is foolish, and hurtful, and cruel, but then it is convenient and it is economical. If the patient sits up, somebody must sit up to watch him: an additional servant would be required. If you fasten him tightly in bed, the whole house may be at ease about him. He may toss and groan, indeed, and suffer fever and thirst and many horrors, but he "can't get out;" so the small and inefficient establishment will not be incommoded. Until the proprietors of private establishments have the courage so to arrange their houses as to enable them to adopt a proper classification of their patients, and to separate the violent from the moderately tranquil, and both from the tranquil and convalescent, these inconveniences must attach to them; until the time comes when the public will bear them no longer.

After the period to which all the preceding remarks apply, the author of the work was removed to a private lunatic asylum, where at first he appears to have been free from personal restraint. But he represents himself as being without occupation, amusement, or variety, &c. When he had read the paper, he had nothing to do but to look out of the window, and when he had looked out of the window till he was tired, he had nothing to do but to read the paper again. The *spirits* were of course soon busy with him; many of them, and of contrary dispositions. When at dinner or at tea, some spirits would order him to eat this and to leave that, and others would order him to eat that and to leave this. Livelier spirits still would order him to waltz round the table; and then to make other patients waltz, though rheumatic and averse to such exercise; and then he would consider himself ordered to wrestle with them. And now an end. Left with patients duller than himself, no book, no amusement, no walk allowed, no attendant present, the sameness produces ennui, ennui leads to restlessness, restlessness finds relief in dancing, and nobody being there to keep this innocent activity within bounds, an attempt is made to excite the rheumatic, the helpless and the unwatched; and this brings on the *regular* crisis, namely, the hasty entry of two keepers, interrupted in their whist perhaps, poor men, or over their afternoon beer, and all heated and affrighted. What do they do? Calm the dancer, and protect the rheumatic, and divert the troubled mind by amusement or by soothing words, or a walk in the fresh air? None of these things. All these modes of succour are neglected at last, just as the prevention of all that calls for them was neglected at first. But the dancer is thrown upon a sofa and forced into a strait-waistcoat. *Cœlum non animum*: he had crossed the channel, but was still in a region where strait-waistcoats flourished, ever ready in the hands of strong men. "*Thus*," he says, "*commenced my second ruin*." Up to this time, he says, he did not know that he was insane. He also complains that he was taken to the private lunatic house without any courtesy or respect, and turned into the house without the ceremony of an introduction. From these observations may be gathered the desirableness of making a lunatic early acquainted with the nature of his malady, in order to abate his self-confidence; and secondly, the importance of treating him with decent respect, adapted to his station in society when in health. It shocks us to see a grisly, ragged man, called up, as it would seem, from labouring work, that we may learn it is or *was* a man of literary fame, or Sir so and so, or a man of higher degree. The dress of such persons should be scrupulously attended to, their occupation regulated by their own taste, and their manners, if possible, kept from sinking into slovenly negligence, by the most unvarying politeness towards them of those who have undertaken to repair their minds and restore them to society. A lunatic, whatever his rank, commonly requires twice as much attention as when he was sane. In a private lunatic house he is usually bereft of two thirds of the attention which he was accustomed to at home. It is quite visionary to talk of preserving this inattention, which is so cheap and convenient, without personal restraints. Tear up strait-waistcoats, take aching hands out of muffs, remove the nightly handcuff and the daily leg-lock, and there must be more servants, more attendants in every asylum, and of a different kind, not merely muscular, but with some intelligence; persons less prone to punish than active to prevent; less disposed to visit dancing with severity than to keep it within salutary bounds; governing, in short, less by the arms and the legs, and more by the brain. This will not be allowed without a struggle. It would be ruinous to the lunatic housekeeper; the present system only ruins the patients.

That the unfortunate author of this work was a most troublesome inmate we learn from his own pages; and that, considering his great restlessness and activity, he was for the most part treated with consideration and forbearance. Yet the description he gives of his being fastened in a sort of restraint-chair every day, with no amusement but to rub and flatten his nose against the wall; and his account of the manner which he was forced into the bath, and occasionally corrected for his strange fancies, sufficiently show that the treatment was extremely defective. Medicine might be given, and proper food, but cheerful recreative exercise seems to have been much neglected, and the closest and most miserable form of complicated restraint, (sitting in a restraint-chair in a strait-waistcoat, and the feet fastened to the floor,) resorted to for a period which no circumstances could render necessary, or even justify. He seems also to have worn the strait-waistcoat in bed, and to have had his hands strapped to the sides of the bedstead, and his feet fastened to the bottom of it. This is in fact, or was, an ordinary method of restraint in old institutions; very convenient to keepers and nurses, but often utterly destructive of the patient. "Fastened thus, lying on my back, I passed my wretched sleepless nights for nearly, if not quite, nine months!" We do not hesitate to state our belief that there never was a case which required this treatment; never a case in which it would not be mischievous. "I had not exercise enough during the day to procure sleep, but I lay exhausted, wearied, agonied, terrified in my spirits, hungering after rest, but unable to procure it." Most true, and terrible, and instructive commentary! No doubt the humane head of the establishment would have put an end to this if he had not been misinformed; but when he was asked by the patient to be allowed to sleep unfastened, the keeper took up his voice, and preventing any words of mercy, "said in an off-hand, impertinent manner, 'O! no, sir, there's no trusting him,' &c. &c., and I remained silent from resentment." This is the usual course of things. First, the head of the house has no very distinct idea what extent of restraint is nightly employed; it is very difficult to ascertain it if nurses and keepers are cunning and cruel; and when a petition *is* put up for liberty, he is then told that he *may* liberate the patient if he chooses, of course; but in that case the house will be upset, or the nurse or keeper must *leave;* and although this leaving is commonly at some subsequent period found to be for the common advantage of patients and of master, the sense of this blessing is not yet awakened, and the idea of losing a vigilant guardian is full of terror. Thus is the lowest tyranny long confirmed; lunatics, like dead men, tell no tales; or if they do mingle with their fanciful complaints some miserable truths, they attract no attention, and occasion the severest keeper no discomposure.

The besetting evil of lunatic asylums is, that every lunatic patient is of necessity made to associate with many other lunatics. If occasionally useful, we cannot but think this practice must often be injurious. The author of the narrative complains much of having been put into a room with eleven others, "certain of them occasionally raving, stamping, bawling, violent madmen." He says that the mental agony, the distress, and the actions of these men, their quarrels with one another, and with the servants, and the rude and cruel manners of the servants occasioned him the greatest distress. Amongst these he was confined hand and foot, *for six months.* He was often left with two or three of them for hours. Indeed, if our object was to make out a strong case against lunatic asylums, we might quote a hundred different passages, of the truth of which we entertain no doubt, and illustrative of the errors which creep, by negligence, into houses, affording in most respects superior accommodations. It is not so much these errors, cruel as some of them are, as the effects of them upon the lunatic's mind, which we wish to point out. These effects show in the most convincing manner how unsalutary, to say the least of them, these yet too common errors are. Here is a book, evidently written before the author's mind had quite regained its health, and yet in which are depicted the most poignant feelings of shame and mortification, occasioned by his finding himself deserted by his family, severed from his friends, deprived of any confidential ear, or of any spiritual comfort and consolation, and exposed, without scruple, question, or explanation, to the most abject treatment in rough and servile hands. We agree with him that a man of his rank, acquirements, and connexions, should have been more considerately managed; and we remark, with pain, that the want of accustomed respects, and of gentlemanly care and attention, added in no trifling degree to the discomforts of his state of trial. He has also recorded many of the ordinary feelings incidental to lunatics of every class; and we think words like the following must fall impressively on the ears of all who have insane persons under their care:

"They who have not been confined in a lunatic asylum, cannot conceive the dreadful and cruel suspense that delay, and not only neglect but the refusal of every-day civilities, together with inattention to just and obvious complaints, occasion. They do not know our wants and fears, because they do not know the danger we are in. They may judge our danger, however, from what these men do; and from what they have done, they may judge what they dare to do; being encompassed, even more than a king, with a hollow impunity, and clothed in the deepest hypocrisy. They who have not endured this confinement do not know how the very suspicion of being a lunatic, coupled with being cut off from all pecuniary resources, shuts the minds of others against sympathy, impedes the proffer of assistance and the exercise of protection, and aught but the show of pity. Neither how it embarrasses the suppliant in his applications for redress, awakens anxiety, excites mistrust, and closes the door of his hopes, whilst he finds himself left defenceless to the sarcasm and persecutions of those he is accusing. .. ........ How much more fearful is such a trial for one who knows that he cannot plead innocence of lunacy; one who, in mind and bodily health, is weak, and thereby more exposed than another to follow a wrong course; exposed to suffer even from treatment which men in sound health might almost laugh at, still more from that which he dreads from having experienced it, and against which he is exasperated; and also, still more liable than the other to lose that gift, lately lost, so dear now, being newly restored to him, the gift of a sound mind and convalescent health; perishing again from want of wholesome communion, shattered by assault, or insidiously undermined."

To us there is a solemn eloquence in these passages, which it would disgrace us to be insensible to. We are by many things convinced that an important era in the management of insanity is at hand. Pinel struck off the chains from all the lunatics in the Bicêtre, in the period of the French Revolution; but the example was well-nigh lost. Tuke, with no less moral courage, established an institution at York, on principles of the purest humanity, and opposed to every prejudice then prevalent and powerful. Beams of accidental light broke in on Bethlem; and riveted fetters fell off,—were even struck off by woman's hands, to be imposed no more. The occupation of lunatics in various works mentioned, and partly put in practice, by Pinel, adopted in Germany, and forcibly recommended by Tuke, was tried to a great extent at Wakefield, and subsequently at Hanwell by the late Sir William Ellis. A great diminution of restraints took place at Nottingham and in other provincial asylums; and their complete disuse was tried with success at Lincoln; an experiment too important not to need the test of a larger institution, which test has been for fifteen months afforded at Hanwell, one of the largest and perhaps, taking it altogether, the finest establishments for the insane in the world. This is an extraordinary change. That it should prove difficult, attempted as it is in buildings constructed solely with a view to the old physical treatment, and filled with officers and attendants to whom the substitution of what may be called the mental in place of the physical treatment is a novelty, full of obvious inconveniences, and promising only remote and refined advantage, is not a matter of surprise. That it should even be interrupted, suspended, delayed for another century would scarcely surprise us. At Lincoln, without any reasonable cause yet communicated to the public, restraints are said to have been resumed by one set of officers after a long discontinuance by others. At Northampton, they have been wholly laid aside. Whatever accident occurs will of course be at first ascribed to the want of straps and chains. Not only every broken window, and every torn blanket, every bruised face, every blow made in sudden passion, and every attempt to climb a wall and see the cheerful world beyond, will be ascribed to the want of manacles, and aggravate and justify the woe of those who weep over the memory of restraints dear to the tyrant heart; but any case of suicide will be set down to it, although suicidal patients were scarcely ever in restraint, and if it should happen that some benevolent and fearless matron or doctor should be killed outright, we may yet behold the triumphant resurrection of all the implements of horrible coercion. Resurrection, do we say? There are still in many *English* Lunatic Asylums miserable objects in cruel gyves and restraints which have been worn *for years;* and in some cases so long that no one survives in the asylum to tell the first occasion of them. These things, we acknowledge, exceed belief, but *we know them to be true.* In the mean time, every hour that passes, but much more every week and every month, supports the humane experiment going on in Middlesex; and we trust that the great Creator whose sun rises and sets on Hanwell will permit it still for many years to rise and set on a house undisturbed by any accident which may be converted into an apology for a revival of cruelty.

We should be extremely sorry to do injustice to Dr. Millingen, whom we take to be a lively author, not much given to serious reflection; and whose work, consisting partly of paradoxes and partly of aphorisms, seems to have been composed for the sake, among other things, of carrying certain satirical foot-notes, and an oddly-conceived preface, scarcely serious, we imagine, or more than a burlesque, by a witty writer of the grand style. But even trivial and jesting objections to the removal of old corruptions are ever welcome to those who wish to preserve them. For this reason we shall here introduce Dr. Millingen's authority in favour of those restraints which we presume he practised when lately at the head of the largest lunatic asylum in Britain. This is his grave and, we are to conclude, well-considered aphorism :

"257. Except in cases of violent mania, restraint is rarely necessary, unless it be to prevent the mischievous idiot and the maniac from destroying property, when gentle restraint is required to prevent them from constantly tearing their clothes and bedding, or breaking the window-panes, or anything they can lay hold of. It may, however, be occasionally employed as a punishment, the dread of which keeps many lunatics in order."

To this sagacious aphorism is appended a recent note, the offspring of circumstances not clearly foreseen, we imagine, when the aphorism was elaborated. This is the note :

"Nothing can be more absurd, speculative, or peculative than the attempt of theoretic visionaries, or candidates for popular praise, to do away with all restraint. Desirable as such a management might be, it can never prevail without much danger to personal security, and a useless waste and dilapidation of property."

The personal character of several portions of Dr. Millingen's work would make it mere affectation in us to deny that we consider these passages to have been modified by his own aggrieved feelings. His preface alludes to "labours and sufferings;" he seems to expect the most virulent hostility in various quarters ; he vents much wrath on justices of the peace ; and in his attacks on matrons of asylums, he seems almost to forget not only what is due to those who undertake those truly heroic as well as philanthropic duties, but what is due from a gallant man to all women—matrons, wives, maids, or widows. Can we wonder, then, that this 257th aphorism is full of contradictions, or that the note to it is also full of contradictions, or even that aphorism 257 is contradicted by an aphorism in the next page, namely, aphorism 260. Aphorism 257

says that, except in cases of violent mania, restraint is rarely necessary; except also in the mischievous idiot and maniac in whom it is *constantly* necessary. The note says, attempts to do away with all restraint are *absurd*, speculative, and *peculative*, but also *desirable;* that they are the attempts of theoretic visionaries, and of candidates for popular praise, leading to much danger to personal security and a useless waste of property, but *desirable.* To complete the measure of contradictions, this great 257th aphorism sanctions the use of restraint as a punishment, "the dread of which keeps many lunatics in order;" yet in the very face of this, on the opposite page, written by the same hand, stands aphorism 260, which propounds with no less authority that "the influence of *fear* will be found to *aggravate* lunacy instead of keeping the patient under proper discipline. The utmost kindness will be found far preferable," &c.

These are but the struggles of constitutional good nature, unaided by steady reflection or earnestness of purpose, with prejudices fomented by vexatious circumstances. It is but right to say that Dr. Millingen objects to strait-waistcoats; but, alas! by what seems a constitutional inconsistency, he lauds sleeves of canvass, by which the arms, he says, "are kept loose alongside of the body;" and he magnifies the leather muffs, "which only," he assures us, "confine the hands;" and are consequently useful with those who, he tells us, "are always tearing or destroying their clothes or bedding;" and who, of course, are always to be in muffs. But of all devices the restraint-chair is his favorite: "the patients have the free use of their arms and hands, and are *only* prevented from roving about, and committing mischief." Who would think that this elegant contrivance was a large deal watch-box, pierced as a close-stool, in and upon which the patient was placed in the morning to remain there till evening, secured in the chair by straps passed through holes in the back of the chair and locked! Rows of these were once to be seen in most asylums. Thirty-five of these were insufficient for Dr. Millingen's enlarged views at Hanwell, and forty-one were at length at his command, in wards where now not one is to be seen. They now form a floor for the carpenter's shop; two or three being preserved as curiosities. The forty-one cost between two and three hundred pounds, which might have been expended with more wholesome effect in wages to a few additional attendants. The expense of these disgusting chairs, now avoided, may at all events form a set-off against that dilapidation of blankets which Dr. Millingen so sincerely appears to lament. And as Dr. Millingen is a gentleman of some pretensions to literature, although hitherto in paths rather curious than useful, we presume to recommend to him a certain Report of the Commissioners for Inquiry concerning Charities, in which at page 350, he will see that in an ancient institution, conducted on the principles of which he so much approves, and containing nothing speculative or peculative, "*the destruction of the blankets is exceedingly great:*" so that something more than strait-waistcoats and muffs, and chairs of restraint, is required for the preservation of bedding.

But one word more justice requires from us. As Dr. Millingen lends the whole weight of his authority, derived from a year's residence at Hanwell, to maintaining a system of personal restraint, it is right to look into the effects of his government when there, in public and uncontradicted documents. After time had been afforded to test the efficacy of Dr. Millingen's restraint system, the Magistrates' Report states (49th Report), that "a relaxed state of discipline and disorder were found to prevail to such an extent as must seriously have injured the character and efficiency of the establishment, had they been allowed to continue." In less than a year afterward, the restraint-system having been wholly discontinued, the magistrates report (52d Report) that "better order and more continued decorum have been maintained; and although occasional outbursts of violence are, from the capricious nature of the disease, unavoidable, they have become rarer, and the whole establishment has less of the characteristic features of a lunatic asylum." To this testimony no reasonable exception can be taken. The present resident physician may be a partisan, or he may look for some credit for his system; he may be vain or prejudiced, or he may conceal the disadvantages of his darling plan. We put him quite out of the question. But we have here the deliberate opinion of the magistrates, who are exposed to obloquy if anything goes wrong; and who, when windows are broken or blankets are torn, have to perform the unpopular task of raising money from the county to repair the damages.

Notwithstanding all this, we are convinced that there is a fund of kindness in Dr. Millingen's disposition: but as his government of Hanwell indicated how little kindness can effect if not regulated by reflection and by systematic habits, so his book shows how little it can oppose the pettiness of personal feeling. There are many passages, especially in the notes to the Aphorisms, the only object of which seems to be to wound some one or other whom he does not love. If quite purged from these defacements, many of the aphorisms might be read with pleasure, and some with advantage. As it is, the work is scarcely worthy of Dr. Millingen's reputation. The oddest fancy haunts Dr. Millingen, namely, that a proposal, made by him, as he says, but, with his usual inconsistency, also ascribed to Dr. Browne, of Dumfries, *that all lunatic establishments should be placed under the control of government*, will subject him to martyrdom; the truth all the time being that his proposition is neither his nor Dr. Browne's; but was made, and printed, and published, and read by all readers, full eleven years ago. Deducting these and other defects, there are many useful observations in the Aphorisms; certainly not all, or many of them, original, but all or most of them worthy of remembrance. With these, however, are intermixed some singular remarks of the author himself. In aphorism 76, it is observed that the complication of paralysis with insanity appears to be much more frequent on the continent than in England, "especially in France;" and this is partly ascribed by Dr. Millingen "to the practice of the French, who carry burthens on their backs and loins instead of their shoulders and head." We are inclined to question the correctness of the statement altogether; particularly as we see that Dr. Millingen states, that out of 1000 patients at Hanwell, only twelve were paralytic; and yet find in a Report of the Hanwell Asylum, made a few months after Dr. Millingen left that institution, that out of 805 cases twenty-three were complicated with paralysis. We are even of opinion that the instances of slight paralysis are in greater proportion; and that the insanity is, in many cases, merely the consequence of a paralyzed condition of part of the brain. The phenomena of these cases are sometimes not very striking; the lesion seems slight, the impairment of mind inconsiderable; but the disease is incurable, and the mind is irretrievably ruined.

In noticing the moral causes of insanity, Dr. Millingen arranges them in the following order: 1, Pride; 2, Fear; 3, Fright; 4, Ambition; 5, Loss of property; 6, Domestic cares. And he immediately and characteristically adds: " Of these, domestic cares are the most active;" so that this class should have been the first instead of the last on the list. The Fifty-first Hanwell Report states that the causes are, " in a great number of instances, referrible to poverty, and the general struggle of the poorer and middle classes for existence."

We are staggered (perhaps in consequence of our ignorance) by aphorism 193: " Whatever may be the cause of the phenomenon, when demonomaniac women are plunged into a bath, and invariably rise to the surface of the water, the case may be considered of an obstinate nature." We should be glad to know on what authority this phenomenon of obstinate resistance to drowning rests. Is it to be found anywhere except in the annals of witchcraft, or in that curious department of literature in which we humbly acknowledge ourselves to be immeasurably Dr. Millingen's inferiors?

The notices of suicide in the 205th aphorism are very interesting:

> "The propensity to commit suicide is more generally observed between the ages of twenty and thirty, and men are more liable to its fatal influence than women, in the proportion of three to one. Men have recourse to the halter, the pistol, or the knife; women to drowning, hanging, starvation, and poison. Most of these unfortunates make use of the ready means afforded to them by their trade: thus a barber will cut his throat with his razor; a surgeon will open an artery with a scalpel; a shoemaker with his leather-cutter; a soldier will use his musket or his pistol, a sportsman his fowling-piece." (p. 205.)

Without vouching for the absolute correctness of all these particulars, we may gather this useful knowledge from their general tenor, that the comparative immunity of public institutions from these distressing accidents is in a great measure to be ascribed to the absence of opportunities suggestive of horrible temptation. The importance, as a preventive of suicide, of promoting a cheerful and hopeful character throughout a lunatic house, will scarcely, we suppose, be disputed; but such a character can never be maintained in houses where restraints are frequently resorted to; and where, consequently, some are punished, and more are frightened and agitated every day.

With the exception of passages relative to restraint, on which we have already animadverted, the section on treatment contains very interesting and even valuable matter. The importance of classification, of employment, of amusement, of kind conduct, of judicious conversation, and the desirableness of avoiding excitement, irritation, or any kind of irregularity, are extremely well enforced. Dr. Millingen entertains perhaps an excessive jealousy of *clerical* interference in an asylum; and states that out of 1000 patients at Hanwell, he had only *four* " who were fit to receive religious consolation in sickness and on the bed of death." Such an observation scarcely seems consistent with the fact, that between two and three hundred lunatics attend the chapel at the asylum at Hanwell every Sunday; yet we believe it to be nearly correct; attendance at chapel is the restoration of an old habit, agreeable to the patient. Private conversation with a minister is not one of their habits, except in the emergencies of sickness; and to propose it, startles and often offends them in other circumstances. We agree with him in thinking that mistaken zeal may do much harm, and that great judgment is required in the sermons preached to lunatics; but are not at all disposed to think permission to partake of the sacrament, "a wild absurdity of bigots;" or that "any patient fit to participate in this service is fit to be discharged."

In the observations on the physical treatment and on the diet of lunatics, will be found many remarks deserving of attention. Dr. Millingen agrees with most of the authorities on the treatment of insanity in considering general bloodletting, seldom admissible. Of local bleeding he thinks highly. When there is "much excitement and increased action of the heart and arteries," *aconite* in fractional doses will, he says, produce a state of calmness more rapidly than detraction of blood. No particulars are given, and there is nothing very lucid in a note to this aphorism, stating, "the endermic inspersion should always be aided by counter-irritation in the intestinal tube;" a sentence as little translated, and as barefacedly "taken from the French," as "a day room *giving* to the garden," and an opening "*practised* in every cell door," and other peculiarities in Dr. Millingen's style. If the endermic *inspersion* refers to the aconite, what do the fractional *doses* mean? And what may uneducated persons like ourselves understand by counter-irritation *in* the intestinal tube, to antagonise these inspersions or these fractional administrations?

The aphorism p. 290, sets forth that "great excitement frequently arises from the want of sleep;" a sentence which we should rather read backwards, saying, "the want of sleep arises frequently from great excitement;" and in this case Dr. Millingen recommends the salt of morphia "according to the endermic method," after slight vesication. Cold applications to the head are advised when there is considerable determination to the brain, and slight pressure upon the carotid arteries. A *yoke* is suggested for this purpose, which we should very much like to see. Its application, it is candidly acknowledged, would be incommodious. Nevertheless, Dr. Millingen assures us that "arterial pressure is a powerful agent in *brainular* excitement, which in insanity, is mostly of a transient nature." Emetics are recommended in cases of melancholia, connected with dyspepsia; and small doses of the tartrate of antimony are mentioned as most efficient in mania. When speaking of purgatives, Dr. Millingen says that croton oil should be prescribed with reserve, as he has "frequently observed increased cerebral excitement with stupor after its administration." In dyspeptic and melancholy cases the compound rhubarb pill, given repeatedly and in small doses, is mentioned as "most effectual;" and castor oil stigmatized as injurious, and sometimes productive of fatal diarrhœa.

We have anxiously read the aphorisms concerning opium, narcotics, and sedatives; being convinced of their efficacy in some cases, of their inutility in others, and of the difficulty of saying, without trial, whether they will do good or harm. Dr. Millingen says opium is injurious in cases of cerebral congestion and great vascular action, "as it will often occasion phrenitis;" and that it may be given with safety when there is "*no* great excitement," &c. This is much too vague. In cases of no great excitement it is not required; and the truth is, that in some cases of great excitement it is strikingly useful. Dr. Millingen prefers the endermic method of applying it in sleepless patients. When given internally, he prefers Battley's solution. His experience of hyoscyamus, belladonna, and conium, is in favour of belladonna. He certainly underrates the hyoscyamus, but it requires to be given in a full dose; two drachms of the tincture will in many cases be found very useful. We partake of Dr. Millingen's unfavorable opinion of digitalis. Its bad effects are soon produced, and benefit seldom attends them. The remarks on counter-irritants are very brief; and the recommendation of the actual cautery in cases of demonomania,—an iron of a globular form, at a white heat, applied to the mastoid process, however ingenious, is not likely often to be practised.

Dr. Millingen's work is concluded by a section on the construction and distribution of public asylums, pervaded by one great fault, that it contemplates the perpetuation of restraints, the discontinuance of which will necessarily suggest many improvements on the old plans of asylums. It is only when an attempt is made to diminish or abolish restraints, that the physician finds the arrangements of the whole house have been modified by habitual dependence on the means without the aid of which he is endeavouring to regulate it. Dr. Millingen justly objects to long galleries, as opposed to classification, particularly when directly communicating with one another: and recommends detached buildings. The inconveniences of detached buildings, are at the same time obvious; but in proportion to the size of a gallery or ward, the chances of disturbance and confusion must always increase. The massive bedsteads praised by Dr. Millingen are useless, at least if made of iron. Massive as they are, they can be moved by a patient, and their coldness is extreme. Wooden beds fastened to the floor, answer every purpose for the refractory; and light iron bedsteads are well suited to other patients. The following axioms are deduced from Hanwell under Dr. Millingen's system:

> .... "On each side of the bed should be slides for passing straps and buckles to keep down the bedding, and *enforce quiet*. To the bedsteads of epileptic patients should be fixed a strap and buckle, to secure one of their hands during the night—a precaution highly necessary, as these unfortunates will *not unfrequently* turn upon their faces and be smothered. Epileptic patients are themselves so well aware of the necessity of this precautionary measure, that they will *often* request the keeper to confine one of their hands at bedtime."

This is merely a revival of Sir William Ellis's notion. At all events, at Hanwell this "highly-necessary" precaution has been discontinued entirely; and of one hundred of these "unfortunates" who used to be strapped to the bed every night in the year, we do not learn that any one has yet petitioned for a renewal of a custom, which neither permitted them to lie comfortably on the right side nor on the left.

M. Lehmann's little work is written in an animated strain. He is not a medical man, and the severe notions of discipline with which he sets out prepare the reader to find him the advocate of terrors; for he censures Dr. Bräunlich for asserting that lunatic patients are not responsible, and therefore ought not to be punished. M. Lehmann thinks the same may be said of a child; and, assuming that punishment is excellent for children (a very disputable notion), he argues that, therefore, it is good for lunatics. Poor as such an argument is, we believe that no better one can be adduced for the practice. In both instances (in that of the helpless lunatic and in that of the defenceless child), a humaner view would take in the possible good effects of prevention. But punishment, like revenge, is sweet; and will not yet be abandoned either in the madhouse, or in the schoolroom, where it prepares subjects for the madhouse, until after many struggles.

Nevertheless, the author elsewhere speaks as a humane man. He takes, indeed, one would sometimes suppose, elevated views of his duty, and eloquently enforces the truths that "love produces love, and esteem, esteem;" that the superintendent is placed in his position for the patients, and the patients are not placed there for him; that these poor creatures are still the image of God, and deserving of sympathy; and, finally, "are unfortunate and not guilty." "The first essential in a superintendent or a keeper," he says, "is to endeavour to acquire the confidence of the patients;" an opinion in which we cordially concur, but the value of which has been surely overlooked by those whose first actions towards the patients are those of violence. He justly points out the importance of studying the character of each patient, in order to effect a useful classification; but, unless patients are treated with the greatest consideration and delicacy on admission, we do not believe that those who have the charge of them ever become acquainted with their real characters. There are bodily maladies which are the joint product of bodily disease and medicine, and we suspect that certain phenomena ascribed to madness have been the joint result of disease and treatment.

The observations of M. Lehmann on the qualifications of the attendants, who are always with the patients, are generally judicious. He thinks it less desirable to give a flat contradiction to a patient's erroneous notions than to attend a little to them, so that the patient may not think himself neglected. Sometimes he has competed with them in their own line; as with a tailor in poetic compositions: it appears that the tailor, although he did not think so highly of M. Lehmann's performance as of his own, was gratified to think that he did not disdain the muses. The necessity of perfect good sense, good temper, and all the other good qualities which, if possible, the guardians of lunatics should possess, is expressively conveyed by M. Lehmann in the maxim, that they should be *soul-sound;* for, although it may not be true that he "who drives fat oxen should himself be fat," it is most true that he who undertakes to regulate the minds of others should be especially careful of his own. He very properly reprobates the carelessness with which abandoned or dissolute characters, fit for nothing else, are thought fit to be intrusted as the keepers of insane patients.

There is surely a providential inconsistency in those who advocate severities. We have seen some edifying examples of this in Dr. Millingen's Aphorisms; and M. Lehmann's pages present others. After finding fault with Dr. Bräunlich for advocating the immunity of lunatics from punishment, he himself asserts that they are unfortunate and not guilty; and yet, immediately afterward, we find him insisting on the importance of "exciting *fear* in the patients, and from the very first." We read the title of this section with incredulity. We thought there might be some advantage in allaying fear, and from the very first. But it is not so. These poor creatures, still God's image, unfortunate and not guilty, are to be improved in their understandings by frightening them out of their wits.

With astonishment and with pain do we read, that, although "love begets love," yet love has its bounds; that kindness is unwholesome for the lunatic; that Jean Paul says, "the rod helps God;" and that Lichtenberg says, "with mad people blows do often more good than all other measures;" aye, and with this epigrammatic reason too, that "through them the soul is forced to knit itself once more to that world from which the cudgels come." Truly, if we admit that the patients are still God's image, we cannot extend that similitude to these physicians. The *antagonizing* principle seems to animate them, and their doctrines are only fit for the inquisition in this world, and for the place of torment in the next. If anything could deepen our disgust on reading such advice to superintendents, it would be that the "English" are praised for carrying it into effect. Too justly, we fear, has Esquirol observed, that the Germans and the English have exhibited the greatest zeal in devising ingenious and cruel bodily restraints.

It is astonishing how much the feelings, when thus perverted, interfere with directness of vision. M. Lehmann seems to have heard how remarkably insane persons are sometimes tranquillized on their introduction to to a lunatic asylum. M. Esquirol observes, that some never exhibit any madness afterwards. The frequent temporary tranquillity induced by the change is dwelt upon in the recent reports of Hanwell. In these instances it has been supposed that kindness, an appearance of consideration and regard, a cheerful building, refreshing gardens, and various other circumstances, may contribute to the end observed. But M. Lehmann, with the fatal obliquity of vision to which we have alluded, attributes all to change, and change alone; and seems to think it is of very little consequence what the change is, provided it is for the worse. He again refers with much facility, it appears, to authorities; quotes Horn with satisfaction, as saying "that a lunatic asylum must be so arranged that a sane person would go mad there," and even speaks with some approbation of Reil's fanciful saying, that "the reception of a lunatic in his asylum should be amid the thunder of cannon; that he should be introduced by night, over a drawbridge, be laid hold of by Moors, thrust into a subterranean dungeon, and put into a bath with eels and other beasts." Upon this M. Lehmann observes, "If this does not bring our patient to attend to what is going on about him, both by eye and ear, I know not what will." We confess that we quote these expressions with a degree of horror. Such doctrines and practice, springing from cold hearts and wild imaginations, at once declare the total unfitness of such men to be intrusted with the care of lunatics. Take this single and simple test of them: If a man has horses that he values, or dogs that he regards, would he intrust them to the care of these *cudgel-players*? Certainly not. He would say, "these are cruel fellows; they will ill-treat my horses and dogs, and ruin their tempers." Can it then be too much lamented, that men disqualified by the brutality of their minds from being the keepers of brutes, should be the guardians of human beings bereft of their protecting reason?

On a former occasion we alluded to M. Leuret's plan for subduing all mental delusions by the *douche*. Did a man proclaim himself an emperor, straightway he was put under the douche-pipe. After a few minutes an opportunity was given to him, by the cessation of the stream, to renounce his imperial claim. If, breathless but unconquered, he was still an emperor, down came the column again and again until he fairly abdicated. It is against this notable discovery that Dr. Blanche directs his pamphlet. The insertion of M. Leuret's Memoir in the volumes of the Royal Academy of Medicine has given it, Dr. Blanche thinks, an importance which entitles it to notice. Twenty years' experience has convinced Dr. Blanche, that, in comparison with the results of a mild and patient treatment of insanity, all violent means are vain. He acknowledges that even Pinel and Esquirol have, in certain circumstances, advocated severity; and he is willing to give to M. Leuret all the advantage of his argument, that if the means be successful we should practise them with firmness; just as the surgeon performs a severe operation to save life. But here lies the whole question. The point to be determined is, whether M. Leuret, ambitious of fame, has published an academical dissertation on a new and cruel method of acting upon an old and cruel principle, or has really found out a method of cure for the most obstinate of maladies. Dr. Blanche quotes from the works of Pinel and Esquirol passages sufficiently expressive of their abhorrence of all cruel methods of control; and he appeals to the practice of M. Pariset of the Salpêtrière, and of M. Desportes in the same institution, and of M. Ferrus at the Bicêtre, as supporting the same views. He then examines the *four* cases on which M. Leuret bases his new doctrine of moral treatment. We agree with Dr. Blanche, that the cases prove rather the effects of a strong impression made on the mind by contradiction, mockery, and contempt, alternated by elaborate kindness, than by the douche. The treatment is certainly not the more humane on that account.

Any document relating to insanity, bearing the signatures of Esquirol and Pariset, is calculated to arrest attention. At the end of Dr. Blanche's work, we find a formal report upon it with these names, the production of M. Pariset, who makes a very ingenious defence for M. Leuret, acquitting him of Dr. Blanche's two principal charges: the affectation of novelty in a method of intimidation, and the general application of the practice as a kind of doctrine. This disposed of, M. Pariset agrees wholly with Dr. Blanche, and shows, with his usual force, the limited range of the means of intimidation in the variety of cases brought under the physician's notice in a lunatic asylum. In an eloquent page of conclusion,

M. Pariset shows that there is " a precept and a maxim" which are to be kept in view in the treatment of insanity. The precept is, to favour the renovation of the organization, by attending to the excretions and to nourishment. The maxim is, to establish with the patients the only authority worthy to be gained; " the only authority to which they yield;" an authority " founded on confidence and respect," and " only to be obtained by justice and goodness." Of the justice of those who have the care of them, he observes that insane people never lose a discerning sense; and goodness, he adds, is itself only justice, and should be conspicuous in every word and action of which the lunatic is the object. Thus alone, he says, will the hearts of the insane be touched; their reason, thus alone, will become docile, and their will controlled. Rigorous conduct, on the other hand, in the physician will degenerate into barbarity in those he employs, and excite in the patients feelings of disgust, of aversion, and a desire to be revenged. These wise and benevolent observations are worthy of M. Pariset, and of him whom he affectionately styles his " master," M. Esquirol.

The Second Report of the Resident Physician at Hanwell, accompanying the Fifty-fifth Report of the Visiting Magistrates of that asylum, has just reached us. It embraces many particulars, and should be read entire. The First Part of it contains numerous tables, showing every circumstance interesting to the statistical enquirer respecting the patients admitted, discharged, cured, or otherwise, and dying in the asylum; and to some of these are annexed several remarks, particularly with reference to those discharged, and to the fatal cases. Part Second of the Report is devoted to an exposition of the general system of management pursued at Hanwell; and commences with the gratifying statement that the use of the strait-waistcoat, the muff, and every kind of strap and chain, and restraint-chair, was discontinued on the 21st of September, 1839; and has not since been resumed. Dr. Conolly briefly traces the progress of improvement in the treatment of lunatics; and shows that in this attempt he has only given a little further extension to the principles avowed in every page of Pinel and Esquirol, both of whom continually regret that every measure of violence becomes a bar to gaining the confidence of the patient. This confidence, Dr. C. maintains, is the key-stone of all moral treatment; an opinion in which he is certainly supported by every respectable authority; and he makes many observations calculated to show that the imposition of restraint always militated strongly against the acquisition of the patient's confidence; and, although regarded as a punishment and a mortification, failed in any degree to promote amendment. The details given concerning the gradual amelioration of conduct in some very old cases, which were considered so violent as to be hopeless, since restraint was wholly discontinued, are extremely interesting; and the reasons alleged for not resorting to restraints in the recent cases are to us quite convincing.

But Dr. Conolly expresses great anxiety that those who determine to try to dispense with bodily restraints, should understand that to take off waistcoats and muffs, and to unfasten leg-locks and hand-straps, is only the commencement of a system which, to be complete, must secure the vigilant and constant co-operation of the whole household, and comprehend careful medical treatment, and a never-wearied patience and philanthropy. A great portion of the second part of the Report is devoted to explain what are termed the substitutes for restraint; and which, as might be expected, do not consist so much of methods for which any claim of novelty is set up, as of the more systematic application of methods already stamped with the approbation of all medical men who have been experienced in the treatment of insanity. Among these, seclusion of the patients is largely dwelt upon, as one of the most important, although like restraint itself very liable to be abused.

It is observed that seclusion is compatible with all the remedial means proper in each case; and that whatever objections apply to it, apply with double force to restraint, which either left the patient able to hurt others, he himself being defenceless, or consigned him to a seclusion embittered by straps and chains. There is, indeed, a wonderful inconsistency in the arguments of those who object to the non-restraint system. They object to seclusion, because restraint is not conjoined with it. They declare that restraint was never abused, was seldom indeed practised anywhere to a great extent; yet they are furious at its discontinuance. They at the same time profess to defend restraints, and yet found a charge against Hanwell that restraint is secretly practised there; a charge so utterly ridiculous as well as false, as applied to an asylum seldom free from visitors for one day in the year, that we do not wonder that the resident physician should have resolved to take no part in a controversy for which few persons can be prepared by experience; and have contented himself with referring to the general state of the asylum itself as the fullest answer to all misrepresentations, and one to which no exception can be taken.

The advantageous effects of the shower-bath are spoken of as established by the experience of them in numerous cases. The douche, without being of greater, or of so much benefit, is thought to be objectionable as more distressing to the patient. Several remarks are made on different medicines recommended in cases of mania; the plan of employment, exercise, and amusement followed at Hanwell is explained; and several directions are given for the selection and duties of the male and female attendants. The great points enforced are the exercise of constant patience and kindness towards the patients; the abandonment of all force or threats, or authoritative language towards them, except in special and rare instances; and the union of the whole household, officers, attendants, and servants, in the great design of regulating every part of their conduct and manner with a view to the comfort and cure of the lunatic inmates of the establishment. Some of these remarks, and the portion of the Report which treats of the difficulties to be encountered in an asylum in which the attempt is made to abolish restraints, point too clearly at the difficulties encountered at Hanwell from officers and attendants accustomed to the daily spectacle of cruel methods of coercion. Feeble and uncertain support appears to have been given to the physician by some of the subordinate officers; and the evils arising from their negligence have occasionally, it seems, been coined into charges against the system. These unpleasant occurrences are very slightly touched upon in the Report; but the newspapers have contained distinct allusions to them in reporting a recent discussion of the magistrates at Clerkenwell. By the same means we learn that even the chaplain is a friend to fetters and manacles; and consequently gives no aid to the physician. In fact, we believe that the physician has received consistent support from one officer alone—the matron, who seems to be a woman of rare endowments, daily exemplifying by her conduct that the most courageous self-devotion in the cell of the poor lunatic, in every circumstance of difficulty and danger, is compatible with all the unfailing kindness required by her peculiar office. Supported by such an efficient assistant, and steadily countenanced by the visiting magistrates, who have shown a great degree of moral courage throughout this experiment, the physician has been enabled to apply the non-restraint system to 1008 patients, without the occurrence of a single accident that restraint would have prevented: and, as a kind of answer to many general and vague objections to his plan, which many may look for who do not care to bewilder themselves with controversy, we think we may point to the following statements of the cures and of the mortality under the two systems, as at least showing that the non-restraint system is not less favorable to the well-doing of the patients than were the strait-waistcoat, the leather-muff, the leg-locks, the hand-straps, the restraint-chairs, and the bed-chains.

PER CENTAGE OF DEATHS AND CURES.

| Year. | Average Number of Patients. | Number of Cures. | Number of Deaths. | Per centage of Cures. | Per centage of Deaths. |
|---|---|---|---|---|---|
| 1831 from May 16 | 295 | 20 | 29 | 6·77 or 10·2 per ann. | 9·83 or 14·98 per ann. |
| 1832 | 427 | 64 | 99 | 14·75 | 23·18 |
| 1833 | 537 | 59 | 77 | 10·98 | 14·33 |
| 1834 | 564 | 48 | 58 | 8·51 | 10·28 |
| 1835 | 580 | 28 | 71 | 4·82 | 12·24 |
| 1836 | 611 | 37 | 65 | 6·05 | 10·63 |
| 1837 | 608 | 27 | 48 | 4·44 | 7·89 |
| 1838 | 662 | 33 | 89 | 4·98 | 13·44 |
| 1839 | 804 | 88 | 78 | 11·19 | 9·7 |
| 1840 to Sept. 30 | 846 | 45 | 50 | 5·31 or 6·64 per ann. | 5·91 or 7·37 per ann. |

